# Clinical Results of Thoracic Aortic Surgery in Patients under Hemodialysis

**DOI:** 10.1186/1749-8090-10-S1-A124

**Published:** 2015-12-16

**Authors:** Makoto Hibino, Akihiko Usui, Hideki Oshima, Yuji Narita, Tomonobu Abe, Yoshimori Araki, Masato Mutsuga, Kazuro Fujimoto

**Affiliations:** 1 Cardiac Surgery, Nagoya University Graduate School of Medicine, Nagoya, Aichi, 466-8550, Japan

## Background/Introduction

The number of patients receiving hemodialysis is increasing year by year, and the number of cases who underwent thoracic aortic surgery is limited but also increases.

## Aims/Objectives

In this study, we clarified the early and mid-term results of open surgery for thoracic aortic aneurysm (TTA) in patients under hemodialysis.

## Method

In consecutive 700 open surgical repair for TTA between January 2002 and October 2014, there are 21 patients under hemodialysis preoperatively. They underwent open repair for aortic root in 2, ascending in 6, arch in 10, descending in 2 and thoracoabdominal aorta in 1. They were 20 male and 1 female with mean age of 63.3 ± 13.3 years and mean duration of hemodialysis was 3.4 ± 7.0 years. Six patients had diabetic nephropathy and 6 patients had history of previous cardiovascular surgery. Nine patients performed under emergency situation. The pathology of aortic lesions included 6 true aneurysms, 4 pseudoaneurysms, 4 acute dissections, 3 chronic dissections, and 2 severely calcified aortas.

## Results

Average intubation duration and average ICU stay was 4.8 ± 7.0 days, and 11.5 ± 13.2 days. Postoperative complications included pneumonias in 11, which resulted in tracheostomy in 6 and reintubations in 7, gastro-intestinal complications in 4 and neurological complications in 3. There were 3 hospital deaths (14%) including 1 early death. There were 7 late deaths including 3 aortic events and 2 cardiac deaths for average 2.5 ± 2.2 years of follow-up The 1-, 3- and 5-year survival rate was 80.3%, 54.2%, 36.2%, respectively.

## Discussion/Conclusion

The rate of emergency surgery for TAA is high in patients under hemodialysis. The rate of respiratory or gastro-intestinal complications is also high. Late deaths and aortic events occurred frequently for follow-up period. However, Surgical repair is the only way to rescue patients with TAA in patients under hemodialysis.

